# Watering Places

**Published:** 1872-07

**Authors:** 


### WATERING PLACES.

Every summer increases the mania among people whose supreme aim it is to be considered fashionable, to visit "watering places," ostensibly to invigorate their failing healths; but generally, to display dress, attend balls, catch beaux, or create a sensation, according to the age, sex, or purse of the venturer.

The fashionable matron, with her lovely but frail daughter, visits Newport or Saratoga, with entirely different notions from that of the plethoric old bachelor, whose gouty extremities are at once indicative of the rich viands with which he has plied himself, at the late suppers in his luxurious club room.

The mother, seeing the waning strength of the daughter, from excessive festivities in the city, hopes to improve her condition by a month or two's hilarity at the so-called health resort; and, at the same time, perchance, find a wealthy and aristocratic husband for her idol of a daughter.

The old chap, with painful joints, and with a desire for seclusion and relief from his pain, is led to believe that his necessities can be accommodated at the same popular place.

Both are disappointed; the mother finds her time entirely occupied in preparing her daughter for the various festivities of day and night, until she becomes weary with the toil. Daughter becomes still more weak and frail, as a result of the constant nervous excitement to which she is subjected; and finally, when unable to rise from her bed, confesses that she longs for her own quiet room at home, where she can rest from unceasing dress, and the noise and confusion of the fashionable ball room.

The old bachelor, in the meantime, plies himself with the all-curing mineral water—drinks a dozen glasses or so, then sits down to his breakfast of ham and eggs, hot cakes, chocolate, etc.; after which he bathes in the famed medicinal waters until dinner; when, at the sound of the gong, he prepares himself for his usual supply of turtle soup, roast beef, salids, pies, cakes, pastry, and his bottle of ale or wine; then follows, as a matter of course, the inevitable cigar, with which he takes his seat upon the verandah, lifts his gouty limbs upon a chair, and wonders why in fury he doesn't grow better;—votes the watering place a humbug—believes it aggravates his disease—calls it an infernal bedlam—is sure he'll die if he remains another day, and resolves to go home and "blow up" his doctor for sending him there—and he does! Poor, innocent, old noodle! He never dreams, nor once imagines that ham, pastry, ale, or cigar, had anything to do with it, not he; and the doctor receives the berating he deserves for sending him to such a place.

The moral of all this is, simply, that a *fashionable* watering resort is *not* the place to go searching for better health. It is a commodity there unknown, and you will find it neither in others nor yet in yourself.

If you are in need of such medication as is afforded through mineral springs, consult your family physician, and let him prescribe the spring, air, and diet necessary to the requirements of your case. Be sure he will not send you to Newport, Saratoga, Long Branch, or other fashionable places of suicide; but rather, to some retired spot, where a calico dress for the lady, and overalls and shirt for the gentleman, will be the recognized, orthodox attire of the place, and where balls, parties, or rich suppers are never known or obtainable. Find such a place this summer—go there and rest from the cares of home and business; when you will return at the expiration of your vacation, refreshed in mind and body, able to run your physical machine in regular American style, for perhaps another year.

